# Radiomics analysis of contrast-enhanced CT predicts lymphovascular invasion and disease outcome in gastric cancer: a preliminary study

**DOI:** 10.1186/s40644-020-00302-5

**Published:** 2020-04-05

**Authors:** Xiaofeng Chen, Zhiqi Yang, Jiada Yang, Yuting Liao, Peipei Pang, Weixiong Fan, Xiangguang Chen

**Affiliations:** 1 Department of Radiology, Meizhou People’s Hospital, Meizhou, Guangdong 514031 People’s Republic of China; 2 GE Healthcare, Guangzhou, Guangdong People’s Republic of China 510623; 3 GE Healthcare, Hangzhou, Zhejiang People’s Republic of China 311100

**Keywords:** Lymphovascular invasion, Clinical outcome, Gastric cancer, Radiomics

## Abstract

**Background:**

To determine whether radiomics features based on contrast-enhanced CT (CECT) can preoperatively predict lymphovascular invasion (LVI) and clinical outcome in gastric cancer (GC) patients.

**Methods:**

In total, 160 surgically resected patients were retrospectively analyzed, and seven predictive models were constructed. Three radiomics predictive models were built from radiomics features based on arterial (A), venous (V) and combination of two phase (A + V) images. Then, three Radscores (A-Radscore, V-Radscore and A + V-Radscore) were obtained. Another four predictive models were constructed by the three Radscores and clinical risk factors through multivariate logistic regression. A nomogram was developed to predict LVI by incorporating A + V-Radscore and clinical risk factors. Kaplan-Meier curve and log-rank test were utilized to analyze the outcome of LVI.

**Results:**

Radiomics related to tumor size and intratumoral inhomogeneity were the top-ranked LVI predicting features. The related Radscores showed significant differences according to LVI status (*P* < 0.01). Univariate logistic analysis identified three clinical features (T stage, N stage and AJCC stage) and three Radscores as LVI predictive factors. The Clinical-Radscore (namely, A + V + C) model that used all these factors showed a higher performance (AUC = 0.856) than the clinical (namely, C, including T stage, N stage and AJCC stage) model (AUC = 0.810) and the A + V-Radscore model (AUC = 0.795) in the train cohort. For patients without LVI and with LVI, the median progression-free survival (PFS) was 11.5 and 8.0 months (*P* < 0.001),and the median OS was 20.2 and 17.0 months (*P* = 0.3), respectively. In the Clinical-Radscore-predicted LVI absent and LVI present groups, the median PFS was 11.0 and 8.0 months (*P* = 0.03), and the median OS was 20.0 and 18.0 months (*P* = 0.05), respectively. N stage, LVI status and Clinical-Radscore-predicted LVI status were associated with disease-specific recurrence or mortality.

**Conclusions:**

Radiomics features based on CECT may serve as potential markers to successfully predict LVI and PFS, but no evidence was found that these features were related to OS. Considering that it is a single central study, multi-center validation studies will be required in the future to verify its clinical feasibility.

## Introduction

Gastric cancer (GC) is the fifth most frequently diagnosed cancer and the third leading cause of cancer death worldwide [1]. In china, GC is the second most common cancer and the second leading cause of cancer death [2]. Surgical resection is the only curative treatment for patients with GC [3]. However, recurrence after curative surgery is as high as 40% [4, 5] and the main cause of high postoperative mortality and low overall survival (OS) rate of GC patients [6]. In addition, the survival rate of patients with early recurrence is lower than that of patients with late recurrence [7]. Therefore, it is crucial to identify GC patients with a high risk for recurrence, especially early recurrence, and to develop individualized treatment plans.

Accumulating evidence has confirmed that the recurrence rate is related to T, N and TNM staging [8, 9]. In addition, lymphovascular invasion (LVI) is another important prognostic factor for GC after surgical treatment [9–11] and is associated with early recurrence [12]. LVI, which refers to lymphatic and/or blood vessel invasion [13], can only be postoperatively diagnosed by histopathology. The preoperative prediction of LVI is remains difficult. Many studies have been focused on the early prediction of LVI. Yin et.al [14]. studied the correlation of the contrast enhancement ratio in multiphase CT images with tumor differentiation and intratumoral microvascular/lymphatic invasion. The study from Ma et.al [13]. showed that LVI is related to the quantitative enhancement parameters of multiphasic dynamic CT. Another study focused on predicting LVI using texture parameters, such as energy and entropy, extracted from contrast-enhanced CT images [15]. However, due to insufficient validation in large studies, these criteria for a preoperative image or radiomics-based diagnosis of LVI in GC are not currently widely recognized. Furthermore, the rich high-dimensional features (radiomics signatures) of contrast-enhanced CT images have not been studied for predicting LVI. Moreover, these studies have not investigated the correlation between clinicopathological risk factors (such as T stage, N stage and LVI status) and disease-specific recurrence and mortality. The significance of radiomics signatures in predicting LVI and the effects of radiomics features and clinicopathological factors on tumor progression need to be further explored.

Therefore, the purposes of this retrospective study were to investigate whether radiomics could be useful in predicting LVI and disease outcome in GC patients and in investigating the added value provided by integrating independent clinicopathological risk factors.

## Materials and methods

### Patients

**Fig. 1:**
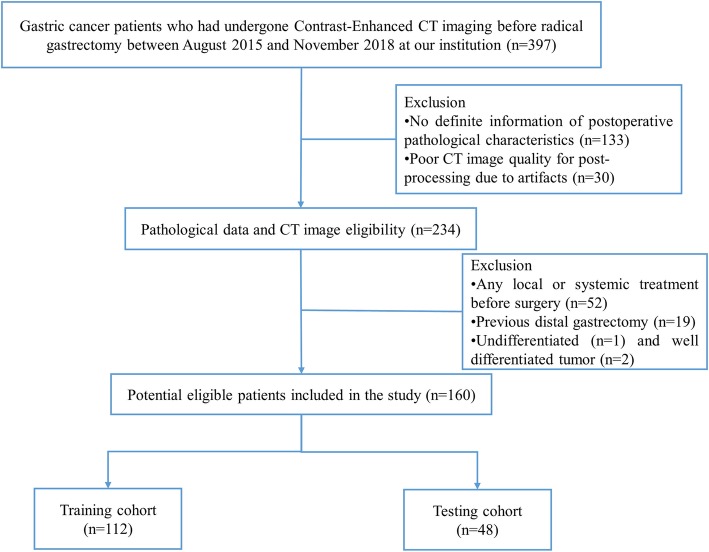
Flow chart showing the patient selection and exclusion

This study was conducted in accordance with the Declaration of Helsinki and was approved by the ethics committee of our hospital, with the requirement for informed consent being waived. From August 2015 to November 2018, 397 GC patients who had undergone radical gastrectomy were gathered and reviewed. The inclusion criteria were as follows: 1) histologically confirmed GC; 2) CECT performed before surgery. The exclusion criteria were as follows: 1) no definite information of postoperative pathological characteristics (*n* = 133); 2) any local or systemic treatment before surgery (*n* = 52); 3) poor CT image quality for postprocessing due to artifacts (*n* = 30); 4) a previous distal gastrectomy (*n* = 19); 5) undifferentiated tumor (*n* = 1) and well-differentiated tumor (*n* = 2). Figure 1 depicts the patient selection process. These patients were randomly divided into a train and test cohort at a rate of 7:3.

The baseline clinicopathological data including age, gender, carcinoembryonic antigen (CEA), cancer antigen 199 (CA199) and tumor node metastasis (TNM) stage, were retrospectively derived from electronic medical records. Laboratory analysis of CEA and CA199 was performed via routine blood tests within 1 week before surgery. According to the normal range used at our institution, the threshold value for CEA and CA199 level was 5 ng/mL and 37.0 U/mL, respectively. The TNM staging was reclassified according to the seventh edition of the Cancer Staging Manual of the American Joint Committee on Cancer (AJCC)/International Union Against Cancer (UICC) staging system.

### Histopathology

All patients underwent surgical treatment within 2 weeks of CECT examination. All surgical specimens were examined by two pathologists, especially in detecting the presence of LVI. The histological parameters ordinarily include tumor differentiation (undifferentiated, poorly, moderately and well differentiated), number of LN metastases, surgical margin and LVI status of the resected tumor. LVI, which refers to as lymphatic and/or blood vessel invasion, was only visible under microscopy.

Finally, a total of 160 patients (age range: 29~87 years; mean age: 62.2 years) served as our study cohort. The patients’ clinicopathological characteristics are presented in Table 1.

**Table 1:** Clinical and histological characteristics of the primary cohort

Variable	LVI- (*n* = 92)	LVI+ (*n* = 68)	*P*^+^	OR (95% CI)	*P* value
Gender			1^+^		
male	63 (68.5)	46 (67.6)		1	
female	29 (31.5)	22 (32.4)		1.04 (0.527~2.03)	0.91
Age			0.5^+^		
≤60	37 (40.2)	32 (47.1)		1	
>60	55 (59.8)	36 (52.9)		0.757 (0.40~1.43)	0.39
T stage			< 0.001^+^		
T1	20 (21.7)	0 (0.0)		/	
T2	11 (12.0)	5 (7.4)		1	
T3	42 (45.0)	31 (47.1)		1.62 (0.51~5.15)	0.411
T4	19 (20.7)	32 (47.1)		3.71 (1.12~12.30)	0.032
N stage			< 0.001^+^		
N0	42 (45.7)	6 (8.8)		1	
N1	21 (22.8)	10 (14.7)		3.33 (1.07~10.4)	0.04
N2	16 (17.4)	18 (26.5)		7.87 (2.65~23.4)	< 0.001
N3	13 (14.1)	34 (50.0)		18.31 (6.29~53.3)	< 0.001
AJCC stage			< 0.001^+^		
I	23 (25)	1 (1.5)		1	
II	35 (38.0)	10 (14.7)		6.57 (0.79~54.8)	0.08
III	33 (35.9)	54 (79.4)		37.64 (4.8~291.8)	< 0.001
IV	1 (1.1)	3 (4.4)		69 (3.36~1415.9)	0.006
Tumor differentiation			0.1^+^		
Poor	48 (52.2)	45 (66.2)		1	
moderate	44 (47.8)	23 (33.8)		0.558 (0.29~1.06)	0.077
CEA			1^+^		
≤ 5 ng/mL	81 (88.0)	59 (86.8)		1	
> 5 ng/mL	11 (12.0)	9 (13.2)		1.12 (0.43~2.89)	0.809
CA199			1^+^		
≤ 37.0 U/mL	81 (88.0)	60 (88.2)		1	
> 37.0 U/mL	11 (12.0)	8 (11.8)		0.98 (0.36~2.57)	0.970
A-Radscore, median (range)	−0.4 (−5.2~1.4)	0.4 (−2.8~1.9)	< 0.001^#^	2.42 (1.62~3.6)	< 0.001
V-Radscore, median (range)	−0.2 (− 26.5~2.1)	0.2 (−2.9~6.1)	< 0.001^#^	1.85 (1.13~3.01)	0.002
A + V-Radscore, median (range)	−0.7 (−5.0~2.1)	0.6 (−3.0~2.1)	< 0.001^#^	2.29 (1.64~3.19)	< 0.001

Note: Unless indicated otherwise, data are the number of tumors with percentages in parentheses. A, V and A + V indicate the predicted model based on arterial phase images, venous phase images and the combination of arterial and venous phase images, respectively.^+^Chi-Square test,^#^ Mann-Whitney *U* test. *LVI* Lymphovascular invasion, *CEA* Carcinoembryonic antigen, *CA199* Cancer antigen 199, *Radscore* Radiomics score, *OR* Odds ratio with univariate test

### Follow-up

According to the follow-up protocol of our institution, the patients were postoperatively followed up with abdomen CT every 6 months for the first 1 year and then annually. Follow-up data were collected from hospital records for patients who were lost during follow-up. The follow-up duration was measured from the time of surgery to the last follow-up date, and information regarding the survival status at the last follow-up was collected. The progression-free survival (PFS) was defined as the time to recurrence at any site, last follow-up, or all-cause death, whichever came first. The overall survival (OS) was defined from the date of surgery to the date of all-cause death, or on 1 March 2019, whichever came first.

### CT image acquisition

All patients signed informed consent forms for CECT examination. The patients were asked to fast from solid food for at least 8 h prior to CT examination and were encouraged to drink 800~1000 ml of water to achieve gastric distension. No anti-cholinergic agent was used. The patients were trained to hold their breath before CT examination. All patients underwent 64-slice multidetector spiral CT (Discovery HD 750, GE Healthcare, Guangdong, China) prior to surgery. All patients were in the supine position and the scan covered the upper or the entire abdomen. The scanning parameters were as follows: tube voltage 120 kVp,180 reference mAs with automated tube current modulation system, slice thickness 5.0 mm, slice interval 5.0 mm, field of view 350 × 350 mm, matrix 512 × 512, rotation time 0.5 s, pitch 0.984 and reconstruction section thickness 1.25 mm. After an intravenous injection of contrast medium (3.0~3.5 ml/s, 1.5 ml/kg, Omnipaque, 350 mg I/ml, GE Healthcare) via a syringe pump, the arterial phase and portal venous phase scans were acquired following delays of 30 s and 60 s, respectively.

### Radiomics feature extraction

Arterial and venous phase CT images (thickness: 1.25 mm) of all patients were downloaded from the picture archiving and communication system and uploaded into the open-source software ITK-SNAP (version 3.6.0, https://itk.org/)**.** According to the literature, focal thickening of at least 6 mm or greater compared with the adjacent gastric wall was determined to be abnormal thickening and cancerous phase [15]. GC presented as thickening of the gastric wall or mass lesions with obvious enhancement on CECT images. The regions of interest (ROI) were manually drawn along the margin of the tumor on each slice of the arterial and venous phase images. Artifacts and the gastric lumen were carefully avoided when drawing the ROI. one tumor was sketched for each patient. The largest lesion was selected if there were multiple lesions in one patient. All layers of the selected tumor were drawn. The segmentation procedure was performed by two readers with more than 10 years of experience. Reader 1 performed tumor segmentations in all 160 patients, and Reader 2 performed tumor segmentations in 30 patients who were randomly selected from the whole cohort to assess inter-reader agreement of the radiomics analysis. When the location was uncertain, the radiologist outlined the ROI according to the pathological or surgical records.

ITK-SNAP was used to generate volumes of interest (VOIs) by drawing 2D ROIs layer-by-layer with a mouse. After the tumors were manually segmented, the arterial phase images, portal venous phase images and corresponding sketched VOIs were imported into AK software [16, 17] (Artificial Intelligence Kit V3.0.0, GE Healthcare, China) for feature extraction. 180 features were extracted from the segmented VOIs of the arterial phase image and portal venous phase image, respectively. The 180 features included histogram parameters (feature numbers = 42), morphological features (feature numbers = 9), gray level co-occurrence matrix (feature numbers = 48), gray level run-length matrix (feature numbers = 70) and gray level size zone matrix (feature numbers = 11). The corresponding formulas and meanings of each feature are detailed in the supplementary materials 1. To remove the unit limits of each feature before the machine learning model is used for LVI classification, the values of each feature for all patients were normalized with Z-scores((x-μ)/σ), where x is the value of feature, μ indicates the average value of this feature for all patients in the cohort, and σ represents the corresponding standard deviation.

To validate the stability of the radiomics features, we assessed the inter-observer agreement of feature extraction using interclass correlation coefficients (ICC) [18]. The features extracted from VOIs delineated by two radiologists (30 patients who were randomly selected from the whole cohort) were utilized to calculate the ICC values, and 180 features were extracted from the segmented VOIs of the arterial phase image and portal venous phase image, respectively. The features with their ICC values greater than 0.75 were selected (139 features for arterial phase image and 43 features for venous phase image). Then, 160 patients with the selected features (139 features for arterial phase image and 43 features for venous phase image) were used for further analysis. The process of feature selection by ICC analysis was shown in supplementary materials 2 (Supp_Figure 1).

### Feature selection and radiomics signature construction

After the ICC selected the repeatable features, Spearman correlation analysis (SPM) combined with the least absolute shrinkage and selection operator (LASSO) method [19] were utilized to select the most useful predictive features in the train cohort. The threshold of the Spearman correlation coefficient was 0.9 to reduce feature redundancy, and the LASSO was used to further select the features with penalty parameter tuning that was conducted by 10-fold cross-validation based on minimum criteria. Predictive models were constructed by multivariable logistic regression with the selected features. A radiomics score (Radscore) was then calculated for each patient via a linear combination of selected features weighted by their respective coefficients in the predictive models, which can be expressed as follows: $\mathrm{Radscore}=\sum \limits_{i=1}^n{C}_i\ast {X}_i+b$, where b is the intercept, *X*_*i*_ is the value of *i* th selected feature and *C*_*i*_ is the coefficient of the *i* th selected feature listed in Table 2.

**Table 2:** Selected radiomics features in A, V and A + V models

Model	Selected features (Total features)	Individual features	Coefficients
A	4 (180)	Intercept	−0.269
		**A**_Max Intensity	−0.581
		**A**_LongRunLowGrayLevelEmphasis_angle135_offset1	−0.440
		**A**_Maximum3DDiameter	0.236
		**A**_SurfaceVolumeRatio	−0.648
V	8 (180)	Intercept	−0.335
		**V**_Percentile5	4.855
		**V**_Quantile0.5	−0.838
		**V**_Quantile0.975	0.215
		**V**_StdDeviation	1.925
		**V**_Uniformity	0.599
		**V**_LongRunHighGrayLevelEmphasis_angle0_offset1	−0.287
		**V**_ShortRunEmphasis_angle45_offset1	0.110
		**V**_IntensityVariability	0.316
A + V	7 (360)	Intercept	−0.257
		**A**_MaxIntensity	−0.566
		**A**_Quantile0.975	−0.206
		**A**_LongRunLowGrayLevelEmphasis_angle135_offset1	−0.412
		**A**_Maximum3DDiameter	0.213
		**A**_SurfaceVolumeRatio	−0.731
		**V**_Quantile0.975	0.612
		**V**_LongRunHighGrayLevelEmphasis_angle0_offset1	−0.154

Note: A, V and A + V indicate the predicted model based on arterial phase images, venous phase images and the combination of arterial phase and venous phase images, respectively. The prefix “**A_**” and “**V_**” represent the features from arterial phase images and venous phase images. For the suffix of “_angle(n)_offset(m)”, n means the rotation angle (i.e., 0 or 45) and m represent the displacement vectors (distance to the neighbor pixel, i.e., 7)

### Development and validation of the predictive model

A univariate regression analyses model of clinical parameters was performed to determine LVI risk factors. The candidate clinical variables were gender, age, T stage, N stage, AJCC stage, tumor differentiation, CEA and CA199 level.

**Fig. 2:**
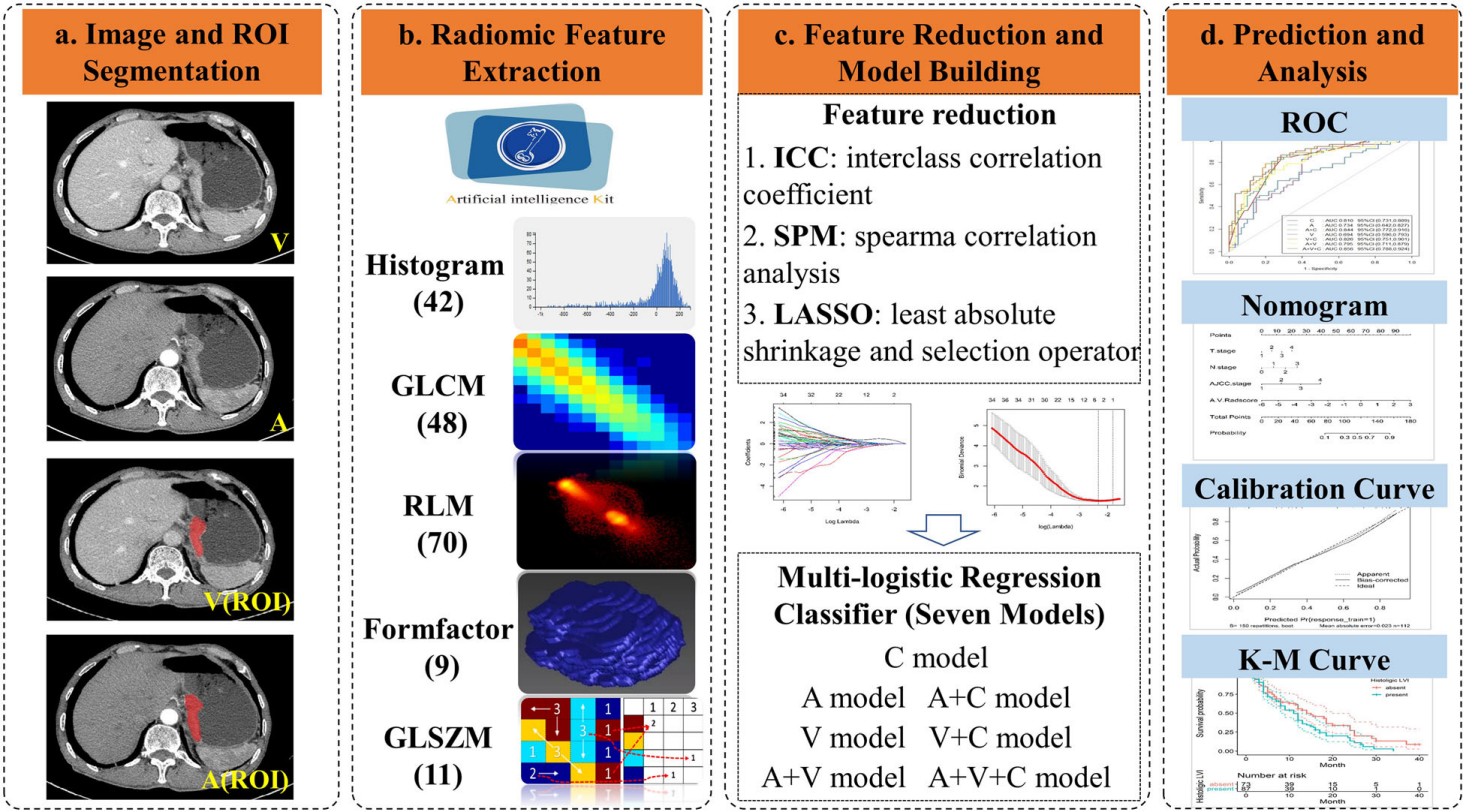
Radiomics prediction pipeline for lymphovascular invasion and outcome

To develop an optimal model, we evaluated 7 models by analyzing the Radscore of arterial (A) phase images, venous (V) phase images, the combination of two phases (A + V) images and then incorporated the independent clinical predictors to build the combination model by multivariable logistic regression analysis. The processes of features selection after ICC analysis and predictive models construction were shown in supplementary materials 2 (Supp_Fig. 2). Furthermore, a radiomics nomogram based on a train cohort was built to provide the clinician with a quantitative tool to predict the individual probability of LVI. The performance of the radiomics nomogram was validated in the test cohort. Calibration curves were plotted to assess the calibration of the radiomics nomogram [20]. The decision curve was conducted to determine the clinical usefulness of the nomogram by quantifying the net benefits at different threshold probabilities in the test cohort. Figure 2 depicts the flowchart of the proposed analysis pipeline described above.

### Statistical analysis

In this study, the patients were divided into groups with LVI and without LVI. The clinicopathological characteristics were compared using the chi-square test for categorical variables and the Mann-Whitney *U* test for continuous variables that were not normally distributed. The independent predictors for LVI status were identified by multivariate logistic regression analysis. In addition, receiver operating characteristic (ROC) curve analyses were performed. The area under the curve (AUC), accuracy, sensitivity and specificity were also calculated. Survival curves were generated with the Kaplan-Meier method and compared by log-rank tests.

Statistical analysis was conducted with R software (Version: 3.6.4, http: www.r-project.org/). The reported statistical significance levels were all two-sided, and the statistical significance level was set to 0.05. The multivariate logistic regression analysis was performed with the “stats” package. Nomogram construction was performed using the “rms” package.

## Results

### Basic clinicopathological characteristics

Statistical analysis of the clinicopathological data of the 160 included patients is listed in Table 1. Out of all patients, 68 were diagnosed with histological LVI in explanted tissue. Patients with LVI had higher T stage, N stage and AJCC stage than those without LVI (*P* < 0.001). The distribution of gender, age, tumor differentiation, CEA and CA199 levels were similar in the two groups (*P* ≥ 0.05). According to univariate analysis, T stage, N stage and AJCC stage were associated with LVI, whereas the age, gender, tumor differentiation, CEA and CA199 levels were not (Table 1).

### Feature selection and radiomics signature construction

Of the features, 180 or 360 texture features in the groups were reduced to form the predictors on the basis of the 112 patients in the train cohort. Table 2 lists the features selected by ICC, SPM and LASSO. According to univariate analysis, patients with LVI had higher radscore than those without LVI (*P* < 0.01, Table 1).

### Nomogram development and validation

**Fig. 3:**
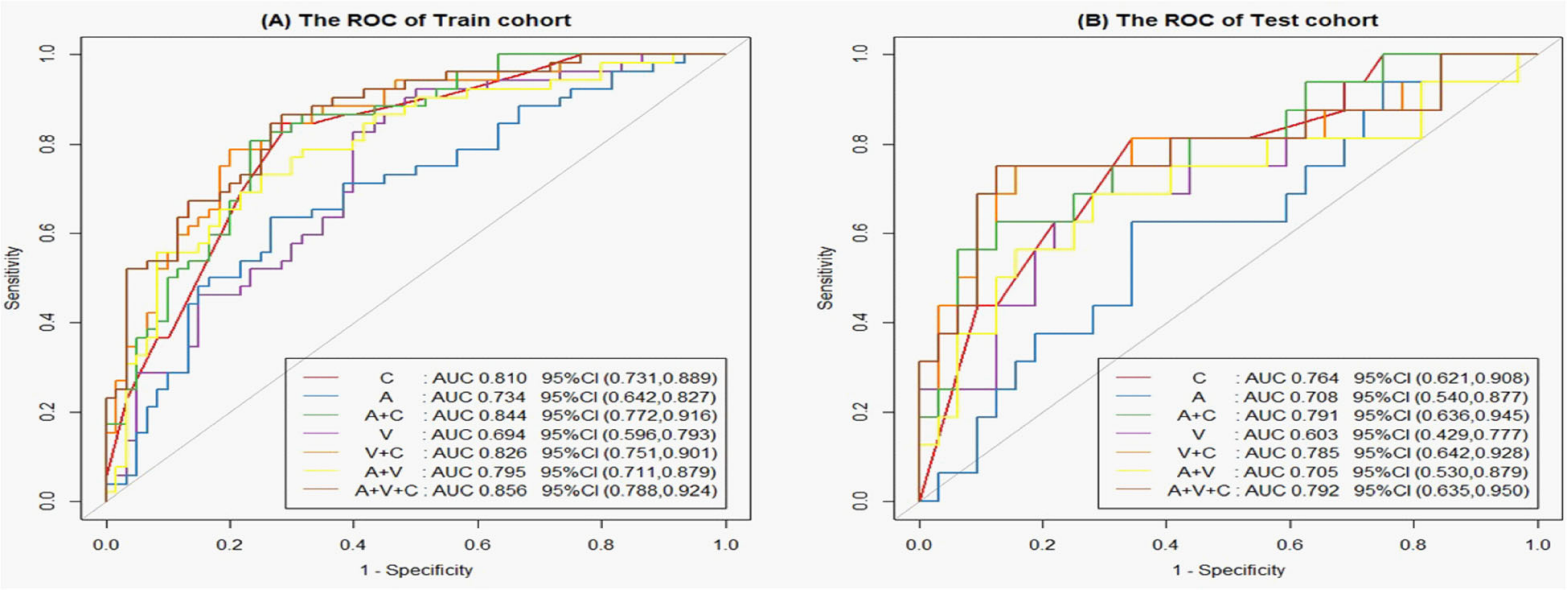
ROC curves of the Radscore, Clinical and Clinical-Radscore for predicting LVI in the train cohort (**a**) and test cohort (**b**)

**Fig. 4:**
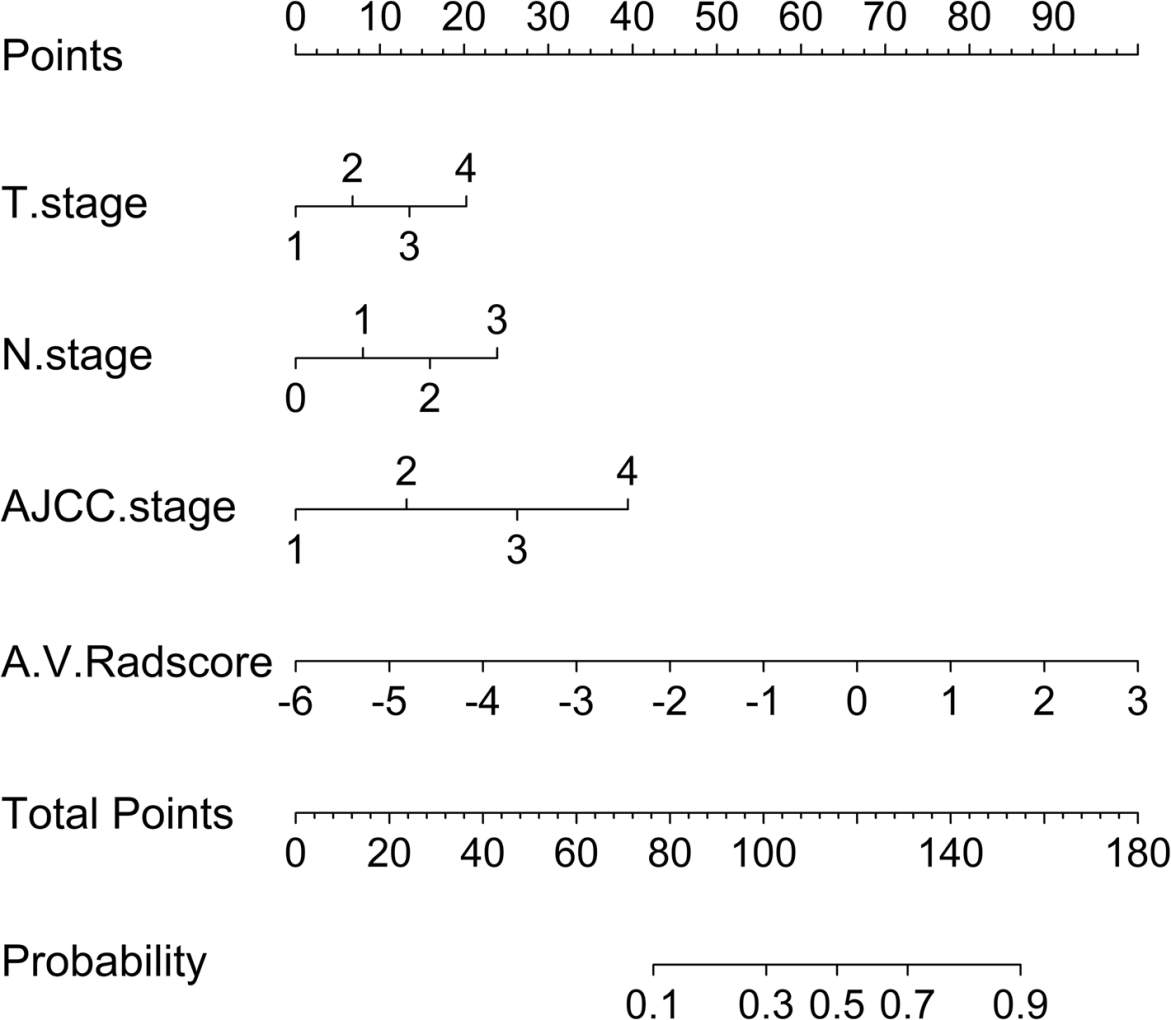
Clinical-Radscore model presented with a nomogram scaled by the proportional regression coefficient of each predictor

**Fig. 5:**
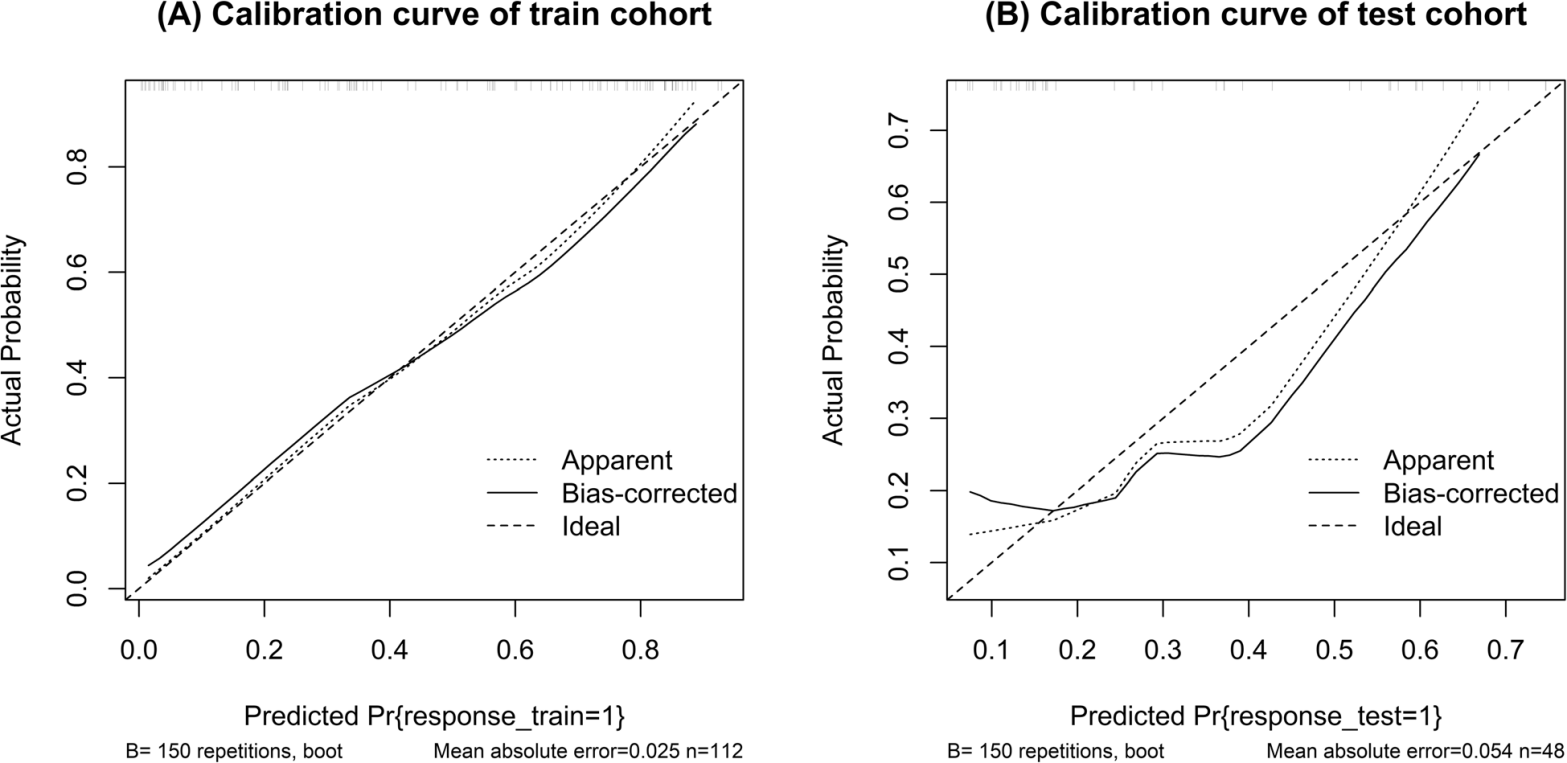
Calibration curve of the Clinical-Radscore model in the train cohort (**a**) and test cohort (**b**)

**Fig. 6:**
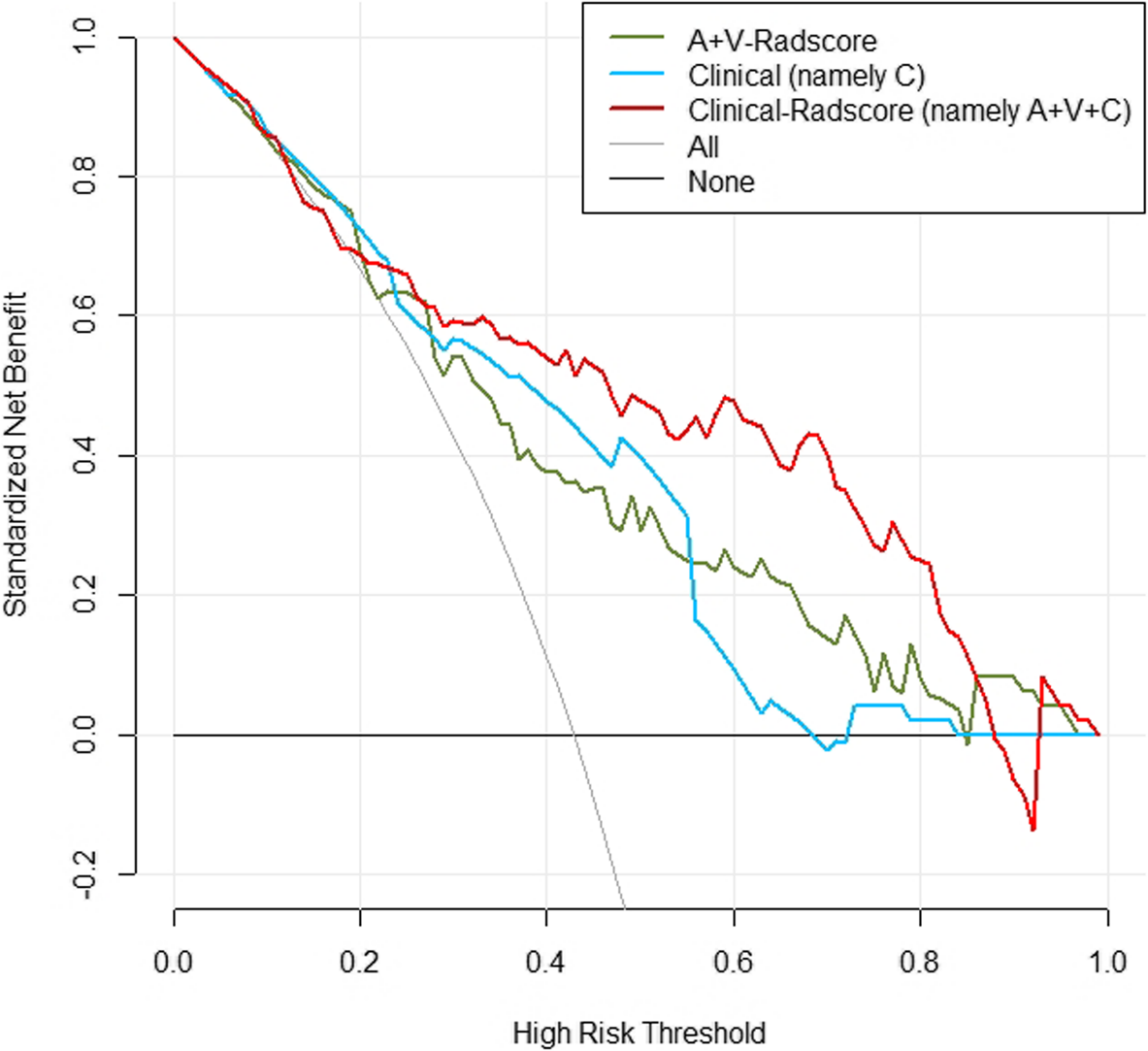
Decision-curve analysis for the A + V-Radscore, Clinical and Clinical-Radscore

The prediction model based on arterial phase images, venous phase images and the combination of two phase images was developed and quantitatively integrated into three Radscores: A-Radscore, V-Radscore and A + V-Radscore. Univariate analyses identified T stage, N stage and AJCC stage as independent predictors (Table 1). Therefore, three Radscores combined with the T stage, N stage and AJCC stage are utilized to develop the predicted model. ROC analyses for the train and test cohort are shown in Table 3 and Fig. 3. The Clinical-Radscore (namely, A + V + C) model yielded a maximum AUC of 0.856 in the train cohort. Therefore, we developed the Clinical-Radscore nomogram (Fig. 4), the calibration curves (Fig. 5) and the decision curve (Fig. 6). The calibration curve of the radiomics nomogram for the probability of LVI demonstrated relatively good agreement between prediction and observation in the train cohort and test cohort. The decision curve showed relatively good performances for the Clinical-Radscore model compared with that for the A + V-Radscore model and the Clinical (namely, C, including T stage, N stage and AJCC stage) model. Across the majority of the range of reasonable threshold probabilities, the decision curve analysis showed that the Clinical-Radscore had a higher overall benefit than the A + V-Radscore and Clinical model.

**Table 3:** Performance of the individualized prediction models

Models	Train cohort				
	AUC	95% CI	Sensitivity	Specificity	Accuracy
T	0.716	0.631~0.802	0.480	0.816	0.660
N	0.783	0.700~0.866	0.750	0.716	0.732
AJCC	0.776	0.701~0.852	0.846	0.666	0.750
C	0.810	0.731~0.889	0.846	0.716	0.776
A	0.734	0.642~0.827	0.826	0.600	0.705
V	0.694	0.596~0.793	0.634	0.733	0.687
A + V	0.795	0.711~0.879	0.730	0.750	0.741
A + C	0.844	0.772~0.916	0.788	0.800	0.794
V + C	0.826	0.751~0.901	0.807	0.766	0.785
A + V + C	0.856	0.788~0.924	0.865	0.716	0.785
Models	Test cohort				
	AUC	95% CI	Sensitivity	Specificity	Accuracy
T	0.661	0.515~0.806	0.437	0.750	0.645
N	0.764	0.624~0.905	0.812	0.625	0.687
AJCC	0.696	0.565~0.827	0.812	0.562	0.645
C	0.764	0.621~0.908	0.812	0.656	0.708
A	0.708	0.540~0.877	0.750	0.468	0.562
V	0.603	0.429~0.777	0.625	0.406	0.479
A + V	0.705	0.530~0.879	0.687	0.656	0.666
A + C	0.791	0.636~0.945	0.750	0.687	0.708
V + C	0.785	0.642~0.928	0.750	0.656	0.687
A + V + C	0.792	0.635~0.950	0.750	0.593	0.645

Note: A, V and A + V indicate the predicted model based on arterial phase images, venous phase images and the combination of two phase images, respectively. T, N and AJCC indicate the predicted model based on T stage, N stage and AJCC stage, respectively. C indicates the predicted model based on the combination of T stage, N stage and AJCC stage. *CI* Confidence Interval

### Predictors of survival

As of 1 March 2019, 160/160 (100%) patients had completed the PFS follow-up, and 155/160 (96.9%) had completed the OS follow-up. The overall recurrence rate was 21.3% (34/160), and the overall death rate was 8.75% (14/160).

**Fig. 7:**
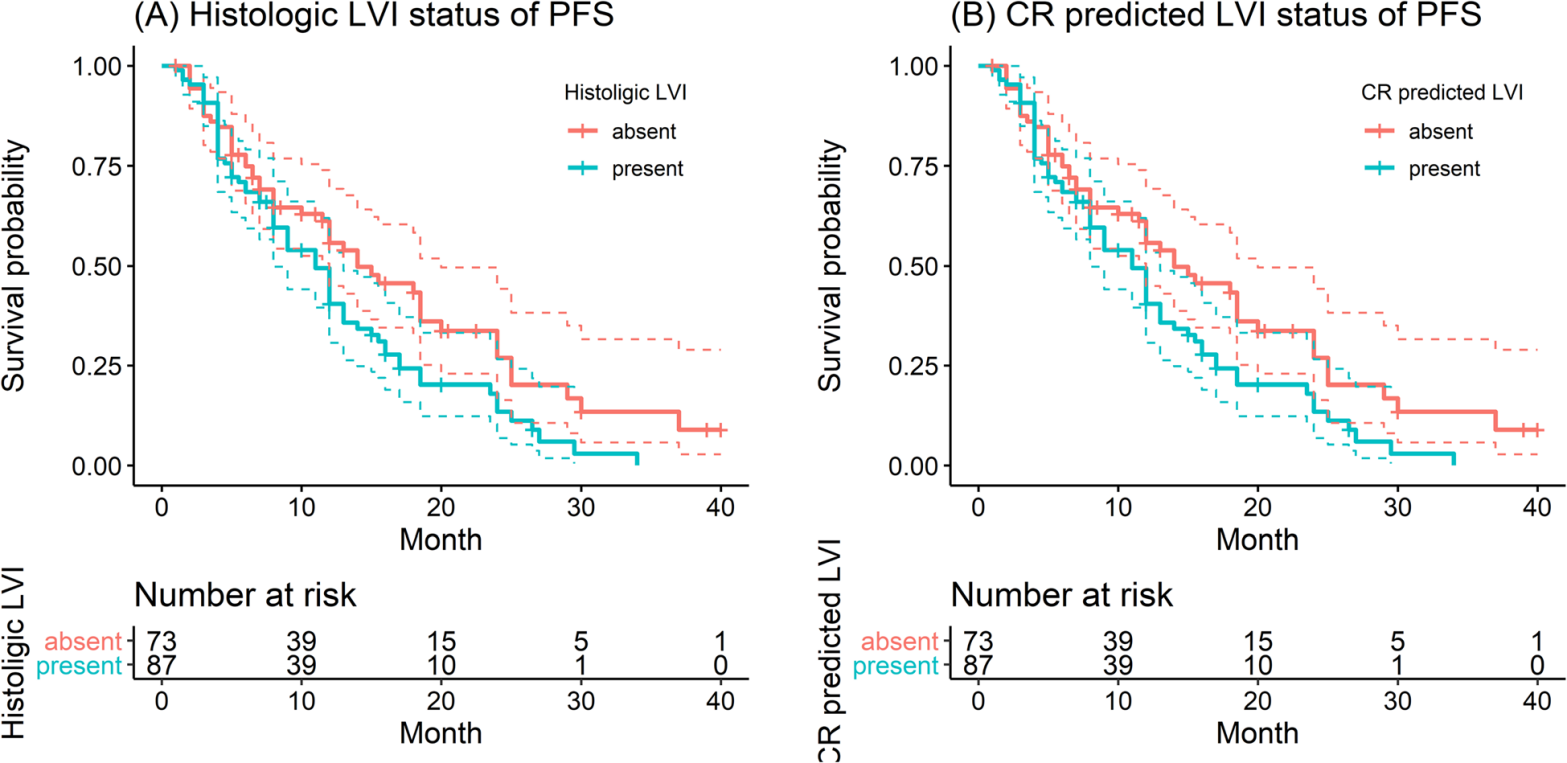
Progression-free survival (PFS) curves scaled by histologic LVI status (**a**) and Clinical-Radscore predicted LVI status (**b**) with Kaplan-Meier analysis

The median PFS of the patients was 9.0 (1.0~40.0) months, and 8.0 (1.5~26.5) months for those with LVI and 11.5 (1.0~40.0) months for those without LVI (log-rank test, *P* < 0.001, Fig. 7a). Similar results were observed in the Clinical-Radscore-predicted (namely, A + V + C) LVI model: The median PFS was 8.0 (1.0~29.5) months for patients with Clinical-Radscore-predicted LVI presence and 11.0 (1.0~40.0) months for those with Clinical-Radscore-predicted LVI absence (log-rank test, *P* = 0.03, Fig. 7b). According to univariate Cox regression analysis, gender (*P* = 0.024), N stage (*P* = 0.006), LVI status (*P* < 0.001) and Clinical-Radscore- predicted LVI status were associated with PFS. Multivariate Cox regression analysis showed that N stage (OR = 1.106; 95% CI: 0.832~1.472), LVI status (OR = 1.595; 95% CI: 1.013~2.511) and Clinical-Radscore-predicted LVI status (OR = 1.208; 95% CI: 0.344~4.238) were independent predictors of disease specific recurrence.

**Fig. 8:**
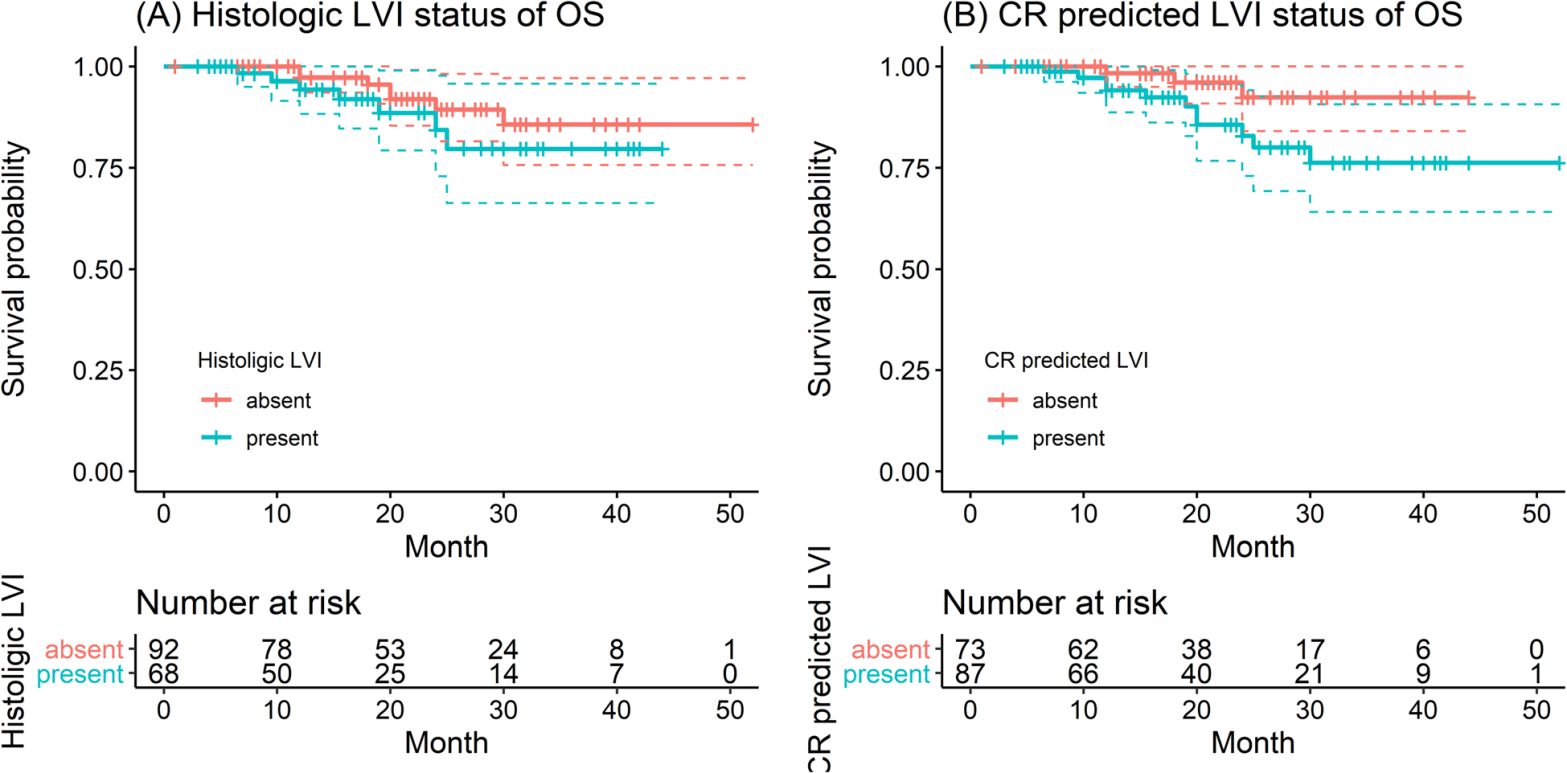
Overall survival (OS) curves scaled by histologic LVI status (**a**) and Clinical-Radscore predicted LVI status (**b**) with Kaplan-Meier analysis

The median OS for all patients was 19.0 (1.0~52.0) months, and specifically, the corresponding values were 17.0 (3.0~44.0) months for those with LVI and 20.2 (1.0~52.0) months for those without LVI (log-rank test, *P* = 0.3, Fig. 8a). The median OS was 18.0 (3.0~52.0) months for those with Clinical-Radscore-predicted LVI presence and 20.0 (1.0~44.0) months for those with Clinical-Radscore-predicted LVI absence (log-rank test, *P* = 0.05, Fig. 8b). According to univariate Cox regression analysis, N stage (*P* = 0.027) and Clinical-Radscore predicted LVI status (*P* = 0.014) were associated with OS. Further, multivariate Cox regression analysis was performed, including N stage and Clinical-Radscore-predicted LVI status as inputs, which showed that N stage (OR = 1.18; 95%CI: 0.515~2.72) and Clinical-Radscore-predicted LVI status (OR = 9.71; 95%CI: 0.337~279.97) were independent predictors of disease specific mortality.

## Discussion

As a common preoperative examination, CECT was an effective tool for differential diagnosis, preoperative assessment, therapeutic and prognostic evaluation in patients with GC [3, 13, 18, 21]. In this study, we built and validated radiomics models based on CECT for the noninvasive, preoperative individualized prediction of histologic LVI status and clinical outcome. We concluded that CECT radiomics features, converted into quantitative Radscore, could be independent predictors of LVI status. The Clinical-Radscore (namely, A + V + C) model integrating clinical features (including T stage, N stage and AJCC stage) and A + V-Radscore (combining arterial and venous phase images) performed well in the prediction of LVI status (ACU = 0.856) and thereby provided an effective tool for clinical decision making. In addition, the Clinical-Radscore predicted LVI status and histological LVI status were associated with disease specific recurrence, suggesting that our findings can play an important role in the clinical treatment of GC.

In contrast to prior studies, we used radiomics features to preoperatively predict LVI status. Among those radiomics features, maximum 3D diameter, standard deviation, uniformity, intensity variability, low gray level emphasis and long run high gray level emphasis were the most important components for predicting histological LVI status. The maximum 3D diameter is measured as the largest pairwise Euclidean distance, where a greater maximum 3D diameter implies a greater tumor size. This finding was consistent with the results of previous studies of hepatocellular carcinoma, which indicated that tumors larger in size had a higher LVI risk [22]. Standard deviation is used to quantify the amount of variation or dispersion of a cohort of data values, where a smaller standard deviation implies a higher vascular invasion risk in GC [15], which is in keeping with our results. Uniformity, intensity variability, low gray level emphasis and long run high gray level emphasis are measures of the homogeneity of the image array, with greater values of these factors implying a greater homogeneity or a larger range of discrete intensity values: This finding is partly in line with previous findings that showed that the lesions with greater homogeneity are more aggressive and drug-insensitive and are associated with worse prognosis [15, 22–25].

In our study, the A + V-Radscore which was based on the combination of arterial and venous phase images achieved an AUC of 0.795 in the prediction of LVI status, which is slightly lower than the value of the Clinical model (namely, C, including T stage, N stage and AJCC stage) predicted model (AUC = 0.810). Because there is a lack of a single highly reliable factor to predict LVI, the radiomics signatures model combining clinical risk factors becomes a viable alternative [11]. By incorporating T stage, N stage and AJCC stage into the prediction model, the overall predictive ability was strong in both the train and test cohort with AUCs of 0.856 and 0.792, respectively. This finding was in excellent agreement with previous findings in the prediction of LVI in hepatocellular carcinoma [26]. Among our risk factors, T stage, N stage and AJCC stage were more important than the Radscores. This result was in accordance with previous results, in which the clinical factors were the top predictor, followed by Radscore [22].

The current AJCC/UICC guidelines do not include LVI as an independent prognostic indicator of GC in the TNM staging system. However, many studies have shown that LVI is an independent risk factor for survival in GC patients [11, 13, 15, 27, 28]. Patients with LVI had been reported to be associated with poorer prognosis, and we also obtained similar results. In our study, the PFS of patients with LVI was significantly worse than that of patients without LVI (median: 8.0 months vs 11.5 months, *P* < 0.001). Similar results in PFS were observed in the Clinical-Radscore-predicted (namely, A + V + C) LVI present and LVI absent groups (median PFS: 8.0 months vs 11.0 months, *P* = 0.03). A potential explanation for the results may be as follows: patients with LVI showed more aggressive disease than those without LVI. However, this finding was not confirmed for OS (median OS of LVI status:20.2 vs 17.0 months, *P* = 0.3; median OS of Clinical-Radscore-predicted LVI status:20 vs 18 months, *P* = 0.05). This result may be because OS is affected by many factors, such as late treatment, other diseases. The multivariate analysis indicated that LVI status, as well as N stage and Clinical-Radscore-predicted LVI status, is an independent prognostic factor in GC patients, which is in accordance with previous results of many studies [11, 29, 30].

Our study has some limitations. First, the Clinical-Radscore-predicted LVI model overestimates the number of LVI+ cases (Histologically LVI+ = 68 vs Clinical-Radscore-predicted LVI + = 87). Although a predictive model with a higher rate of false-positives leads to a better prognosis than that with a higher rate of false-negatives. A high false-positive rate remains a major limitation of this work. More machine learning or deep learning methods will be utilized to further improve the accuracy of the predictions in future research. Second, our model only included traditional clinicopathological factors. The inclusion of additional variables, such as qualitative and quantitative features (e.g. tumor volumes, tumor contrast enhancement ratios, tumor-to-spleen ratio) that can be routinely extracted from multiphasic dynamic CT, may improve the prediction accuracy of LVI status and survival. This potential effect warrants future research. Finally, the study results were assessed in a single institution which has some inherent limitations. The generalization of the results in multi-center should be required in the future studies.

## Conclusion

Radiomics features based on CECT may serve as potential markers to successfully predict LVI and PFS, but no evidence was found that these features were related to OS. Considering that it is a single central study, multi-center validation studies will be required in the future to verify its clinical feasibility.

## Supplementary information

**Additional file 1.**

## Data Availability

The data cohorts used and/or analyzed during the present study are available from the corresponding author on reasonable request.

## Abbreviations

CECT: Contrast-enhanced CT
LVI: Lymphovascular invasion
GC: Gastric cancer
Radscore: Radiomics scores
A: The predicted model based on arterial phase images
V: The predicted model based on venous phase images
A + V: The predicted model based on a the combination of arterial and venous phase images
C: The predicted model based on a the combination of T stage, N stage and AJCC stage
ROC: Receiver operating characteristic
SPM: Spearman correlation analysis
LASSO: Least absolute shrinkage and selection operator
PFS: Progression free survival
OS: Overall survival
CEA: Carcinoembryonic antigen
CA199: Cancer antigen 199
