# Supplementary material for: Radiomics analysis of contrast-enhanced CT predicts lymphovascular invasion and disease outcome in gastric cancer: a preliminary study
Source: Cancer Imaging. 2020 Apr 5;20:24. doi: 10.1186/s40644-020-00302-5 (PMC7132895; doi:10.1186/s40644-020-00302-5)
Supplement: Supplementary file 1 — Additional file 1. [file 40644_2020_302_MOESM1_ESM.docx]

**Supplementary Materials2**

**CAIG-D-19-00289R1**

**Title: Radiomics analysis of contrast-enhanced CT predicts lymphovascular invasion and disease outcome in gastric cancer**

Supp_Figure 1 shows the process of feature selection by interclass correlation coefficients (ICC) analysis in arterial and venous phase image. In this work, the features extracted from VOIs delineated by two radiologists (30 patients who were randomly selected from the whole cohort) were utilized to calculate the ICC values, 180 features were extracted from the segmented VOIs of the arterial phase image and portal venous phase image, respectively. The features with their ICC value greater than 0.75 were selected (139 features for arterial phase image and 43 features for venous phase image).Then, the 160 patients with the selected features (139 features for arterial phase image and 43 features for venous phase image) were used for further analysis.

**
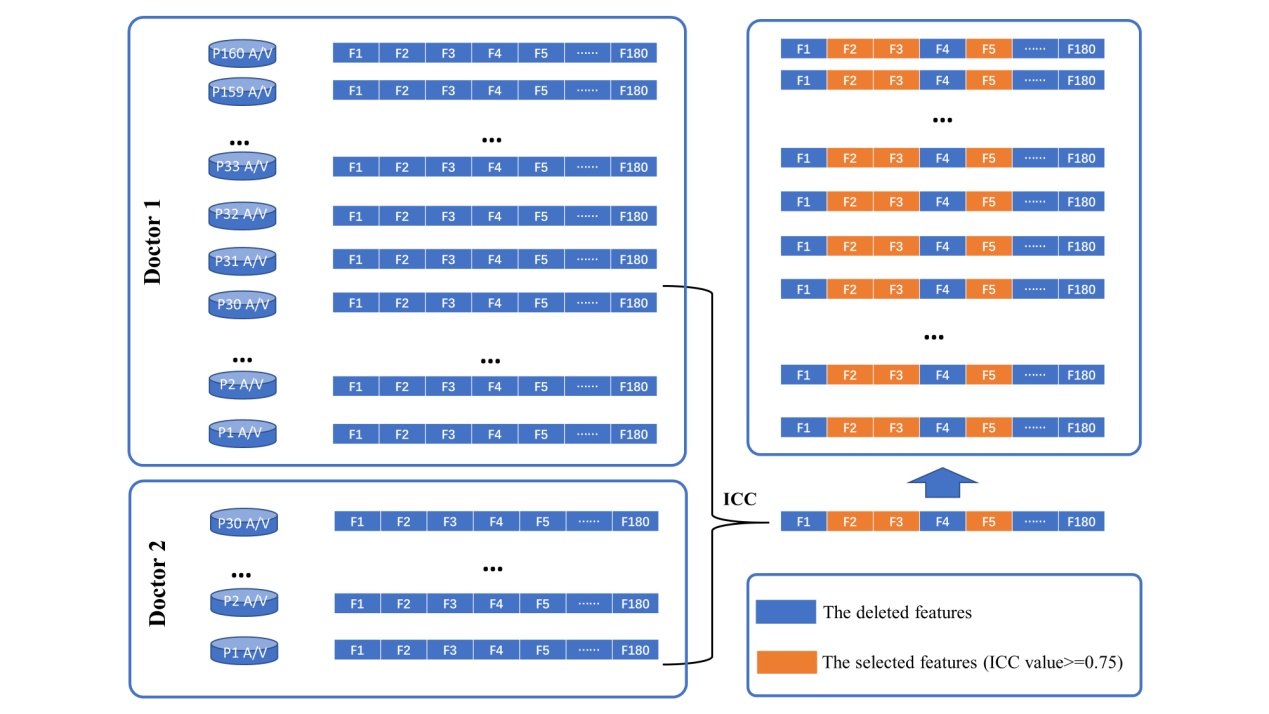
**

Supp_Figure 1 Flow chart of feature selection by ICC analysis in arterial and venous phase image. A/V represents arterial/venous phase image; P1, P2, …, P160 represents the patients; F1, F2, …, F180 represents the features. ICC: Interclass Correlation Coefficients

Supp_Figure 2 shows the flow chart of features selection after ICC analysis and predictive model construction.139 features for arterial phase image, 43 features for venous phase image and 4 clinical features with *P*<0.05 (Table1) are used to constructed 7 predictive models. SPM and LASSO are used for further feature selection. Multi-logistic regression is utilized for model construction.


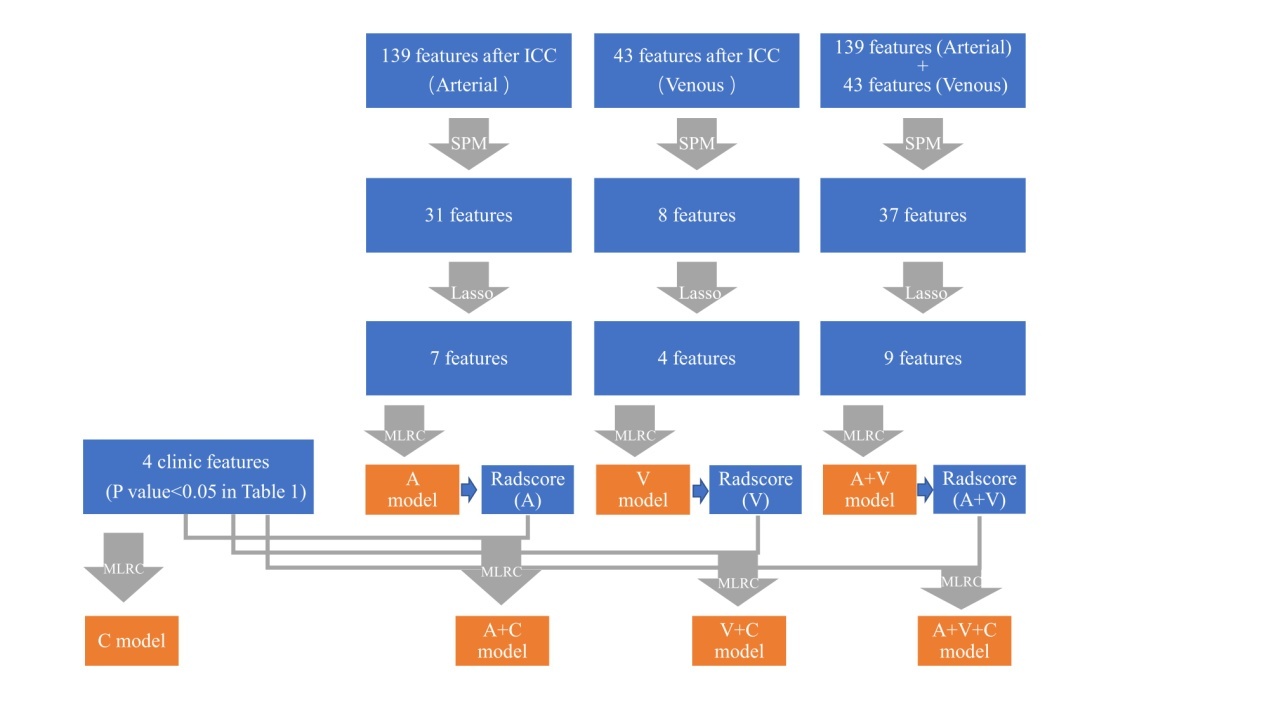


Supp_Figure 2 Flow chart of feature selection after ICC analysis and predictive model construction. ICC: Interclass Correlation Coefficients; SPM: Spearman correlation analysis; LASSO: Least absolute shrinkage and selection operator; MLRC: Multi-logistic Regression Classifier.
